# Supplementary material for: Synthesis, Biological Activity and Molecular Docking of Chimeric Peptides Targeting Opioid and NOP Receptors
Source: Int J Mol Sci. 2022 Oct 21;23(20):12700. doi: 10.3390/ijms232012700 (PMC9604311; doi:10.3390/ijms232012700)
Supplement: Supplementary file 1 [file ijms-23-12700-s001.zip › ijms-1959800-supplementary.pdf]

Supporting Information

Synthesis, biological activity and molecular docking of chimeric peptides targeting opioid and NOP receptors

Karol Wtorek[1], Alessia Ghidini[2], Luca Gentilucci[2], Anna Adamska-Bartłomiejczyk[1], Justyna Piekielna-Ciesielska[1], Chiara Ruzza[3], Chiara Sturaro[3], Girolamo Calo'[4], Stefano Pieretti[5], Alicja Kluczyk[6], John McDonald[7], David G. Lambert[7] and Anna Janecka[1]

[1]*Department of Biomolecular Chemistry, Medical University of Lodz, Mazowiecka 6/8, 92-215 Lodz, Poland*
[2]*Department of Chemistry, "G. Ciamician", University of Bologna, Via Selmi 2, 40126, Bologna, Italy.*
[3]*Department of Neuroscience and Rehabilitation, University of Ferrara, Ferrara, Italy*
[4]*Department of Pharmaceutical and Pharmacological Sciences, University of Padova, Largo Meneghetti 2, 35131 Padova, Italy*
[5]*Istituto Superiore di Sanità, National Center for Drug Research and Evaluation, Rome, Italy*
[6]*Faculty of Chemistry, University of Wroclaw, 50-383 Wroclaw, Poland*
[7]*Department of Cardiovascular Sciences, University of Leicester, Anaesthesia, Critical Care and Pain Management, Leicester Royal Infirmary, LE27LX Leicester, UK.*

Contents

Table S1. Physicochemical characterization of **KW-495** and **KW-496** …..…………… p. S2

Figure S1. High resolution mass spectrum of **KW-495** .... p. S3

Figure S2. High resolution mass spectrum of **KW-496** ……….................................... p. S4

Figure S3,S4. Concentration-response curves in the functional assay .... p. S5

Figure S5. Ligand-receptor interactions for **KW-495**/KOP and **KW-496**/KOP ………. p. S7

Figure S6. Overlay of **KW-495** and **KW-496** into KOP .... p. S8

Figure S7. Ligand-receptor interactions for **KW-495**/NOP and **KW-496**/NOP ………. p. S8

Table S1. Physicochemical characterization of hybrid analogs.

No.	Sequence	Formula	m/z $[M + nH]^{n+}$		HPLC t_R [b] [min]
			Calcd.	Obsd. [a]	
**KW-495**	Tyr-c[D-Lys-Phe-Phe-Asp]-Arg-Tyr-Tyr-Arg-Ile-Lys-NH_2	$C_{79}H_{110}N_{20}O_{15}$	n = 2; 790.4303<br>n = 3; 527.2893<br>n = 4; 395.7188	790.4345<br>527.2893<br>395.7182	14.138
**KW-496**	Tyr-c[D-Lys-Phe-Phe-Asp]-Gly_3-Arg-Tyr-Tyr-Arg-Ile-Lys-NH_2	$C_{85}H_{119}N_{23}O_{18}$	n = 3; 584.3107<br>n = 4; 438.4849	584.3126<br>438.4855	13.643

[a] Observed by ESI MS^+ ionization. Mass spectra of peptides were recorded using Shimadzu IT-TOF mass spectrometer equipped with ESI ion source and operated in positive ion mode.
[b] RP-HPLC was performed on a Vydac C_{18} column (5 µm, 4.6 × 250 mm) using the solvent system of 0.1% TFA in water (A) and 80% acetonitrile in water containing 0.1% TFA (B) and a linear gradient of 0–100% solvent B over 50 min, with a flow rate of 1 mL/min.

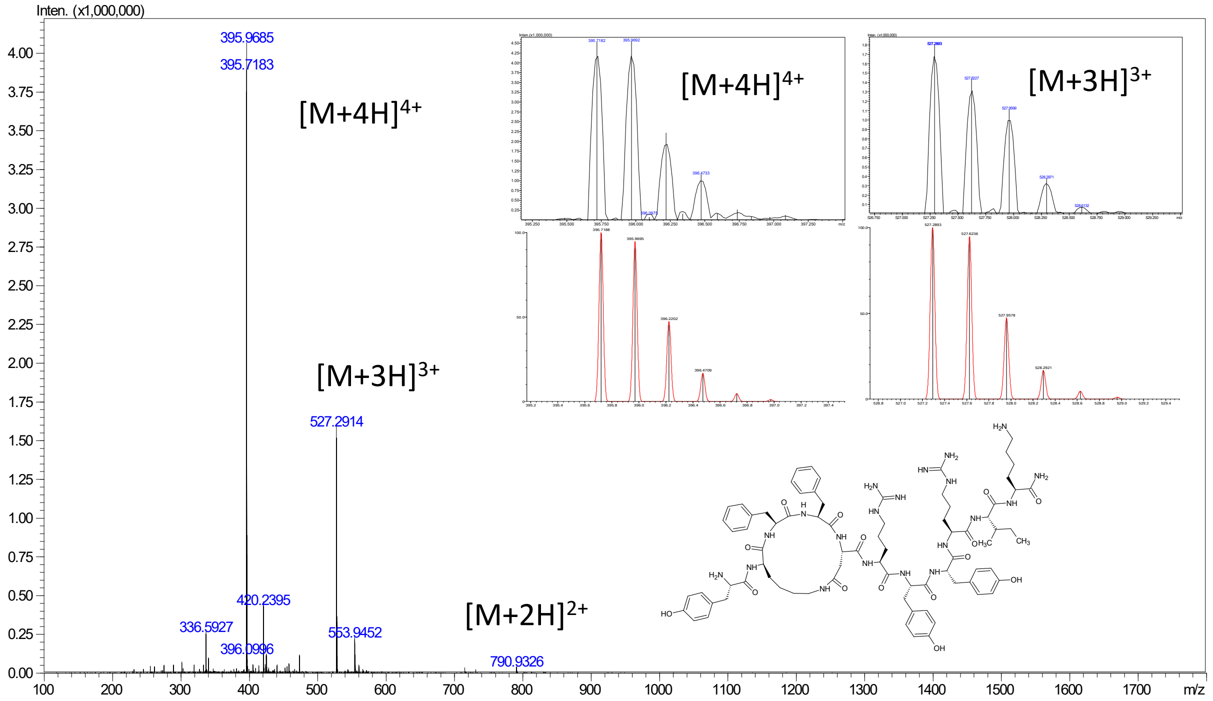

Figure S1. Analysis of peptide H-Tyr-c[D-Lys-Phe-Phe-Asp]-Arg-Tyr-Tyr-Arg-Ile-Lys-NH_2 (**KW-495**)

High resolution MS analysis. In inset, fragments of the experimental spectrum (black, top panels) are compared with the simulated isotopic profiles (red, bottom panels)calculated for the expected molecular formula of protonated species $[M+4H]^{4+}$ and $[M+3H]^{3+}$.

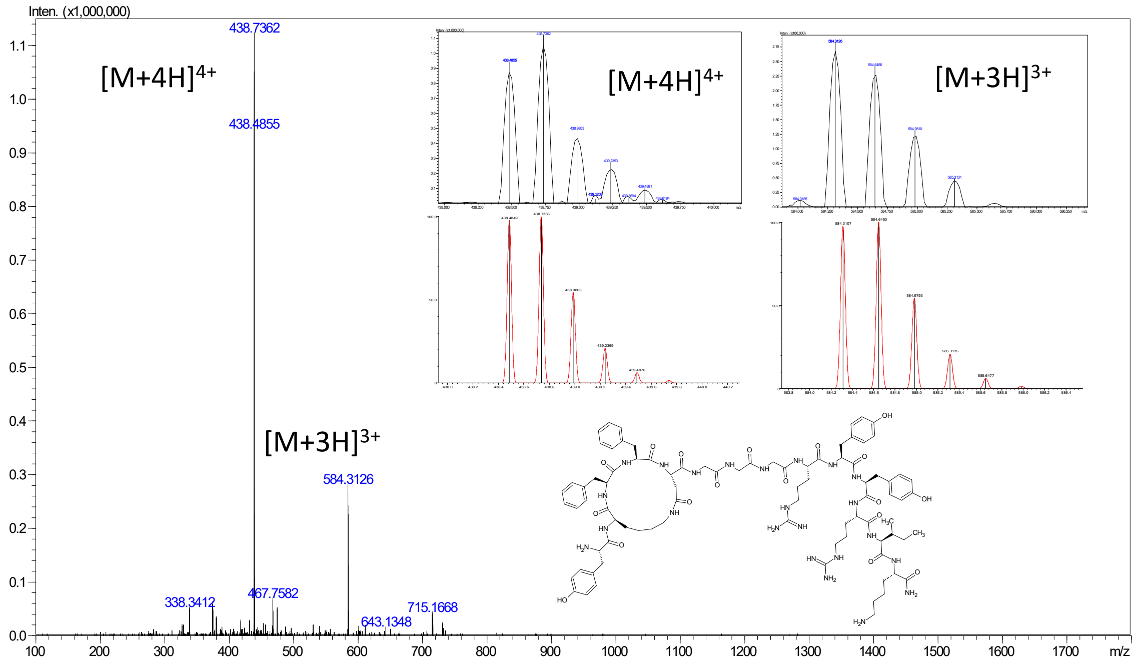

Figure S2. Analysis of peptide H-Tyr-c[D-Lys-Phe-Phe-Asp]-Gly-Gly-Gly-Arg-Tyr-Tyr-Arg-Ile-Lys-NH_2 (**KW-496**)

High resolution MS analysis. In inset, fragments of the experimental spectrum (black, top panels) are compared with the simulated isotopic profiles (red, bottom panels) calculated for the expected molecular formula of protonated species $[M+4H]^{4+}$ and $[M+3H]^{3+}$.

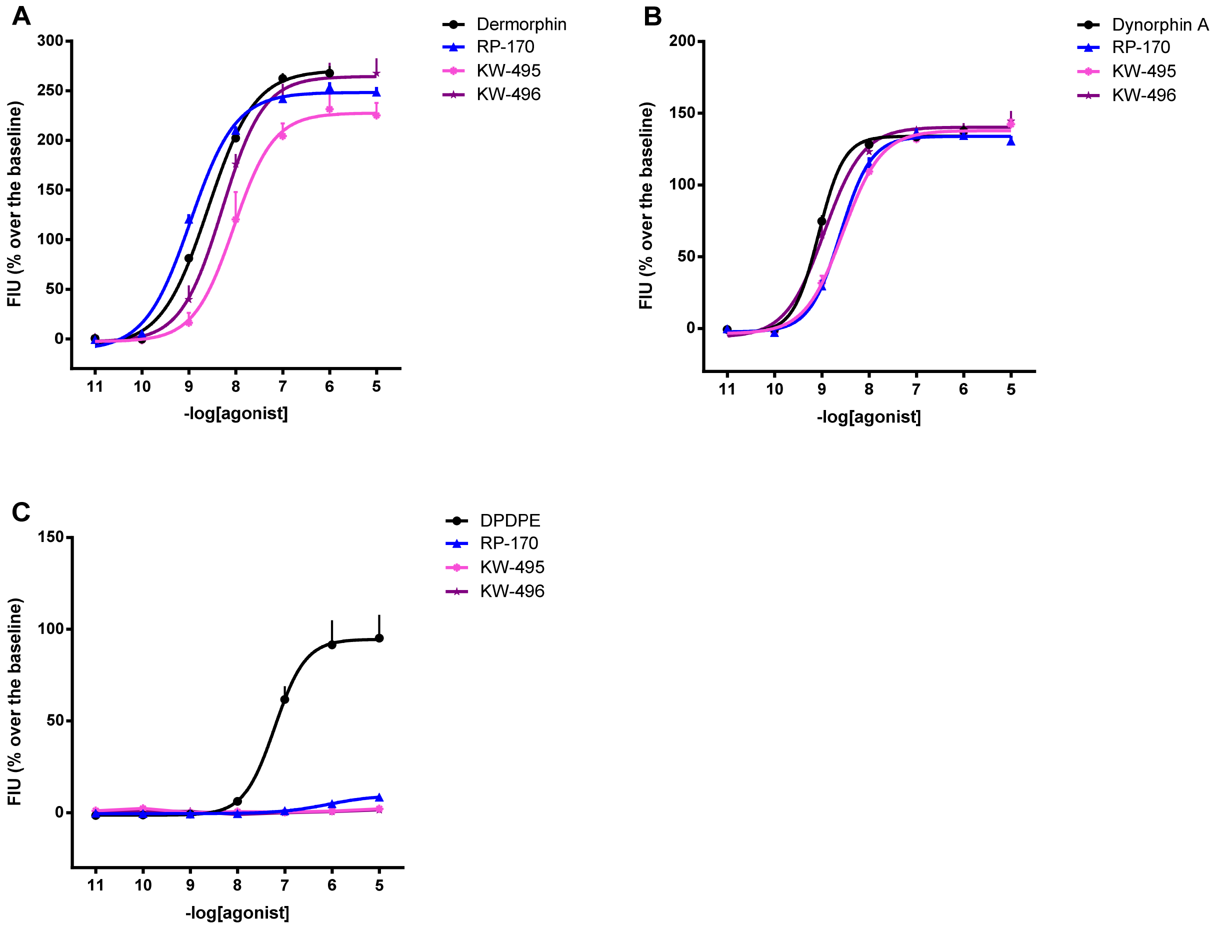

Figure S3. Concentration-response curves to reference agonists and tested peptides in calcium mobilization experiments performed in CHO cells stably co-expressing the human MOP (Panel **A**) or KOP (Panel **B**) and the C-terminally modified $G\alpha_{qi5}$ and CHO cells co-expressing DOP (Panel **C**) and the $G\alpha_{qG66Di5}$ protein. Data are expressed as the mean ± SEM of at least 4 separate experiments performed in duplicate.

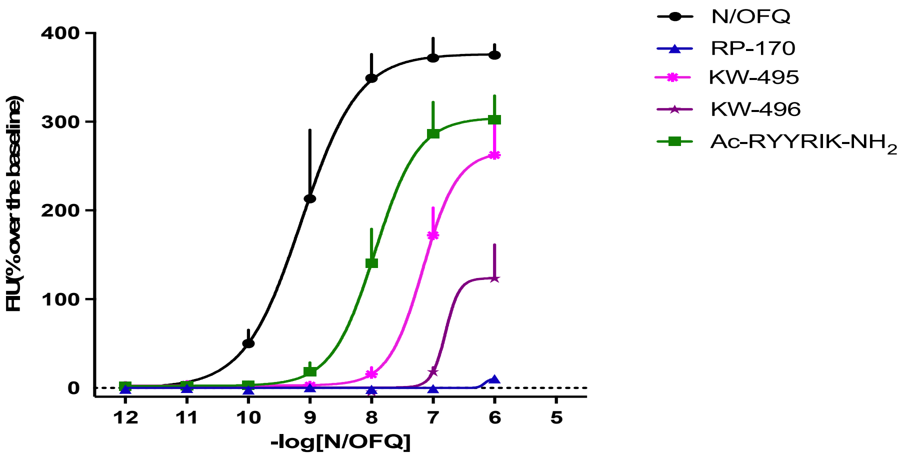

Figure S4. Concentration-response curves to reference agonist N/OFQ and tested peptides in calcium mobilization experiments performed in CHO cells stably co-expressing the human NOP and the C-terminally modified Gα_{qi5} protein. Data are expressed as the mean ± SEM of at least 4 separate experiments performed in duplicate.

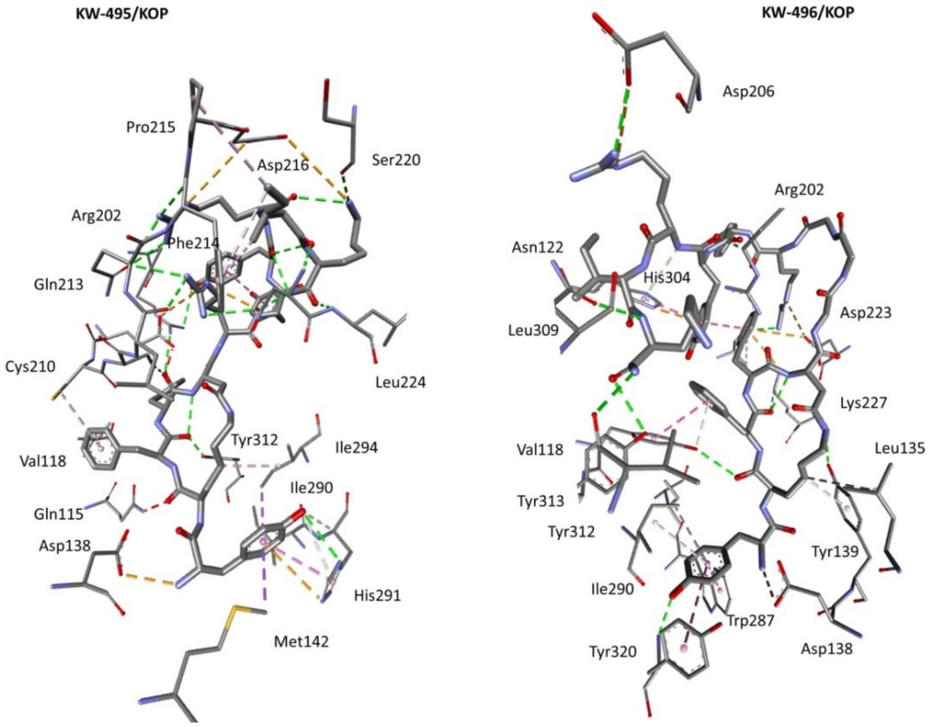

Figure S5. Relevant ligand-receptor interactions obtained with Biovia Discovery Studio 2016 for **KW-495**/KOP and **KW-496**/KOP. Specific interactions not discussed in the main text are the resumed here. For both cyclopeptides, there is an intramolecular H-bond between Asp^5NH and Phe^3C=O (γ-turn). For **KW-495**, Tyr^1-c[D-Lys-Phe-Phe-Asp]-Arg^6-Tyr-Tyr-Arg-Ile-Lys^{11}-NH_2, the cyclopeptide ring is stabilized in its position by a H-bond between D-Lys^2C=O and Gln115; Phe^3 aryl ring is in contact with Val118 and Cys210; Phe^4C=O is H-bonded to Arg202; the methylenes of D-Lys^2 are in contact with the side chain of Ile294. There is an intramolecular interaction between Tyr^7 and Tyr^8 (pi-pi), and between Lys^{11}NHζ and Arg^9C=O. For **KW-496**, Tyr^1-c[D-Lys-Phe^3-Phe^4-Asp]-Gly_3-Arg-Tyr-Tyr-Arg-Ile-Lys-NH_2, the cyclopeptide body is kept in place by Tyr139 (H-bond with cyclopeptide LysNHζ), Tyr312 (H-bond with LysC=O, and pi-pi stacking with Phe^3), Leu135 (hydrophobic interaction with Lys CH_2s), Lys 227 (cation-pi with Phe^4), Asp223 (anion-pi with Phe^4); other interactions involve Arg202(EL-2)-Tyr^{10} (C-H), His204 (EL-2)-Tyr^{11} (pi-pi), His304 (EL-3)-imidazole-Ile^{13}C=O, His304-Arg^{12} (H-pi), Leu309-Ile^{13} (hydrophobic). There is one intramolecular interaction, between the aryl side chains of Phe^4 and Tyr^{11} (pi-pi stacking). Receptor residue side chains are rendered in thick lines and the ligands in sticks; Ile208 has been removed for clarity; C is rendered in grey, N in blue, and O in red. Salt brides are rendered as dashed lines, dashed green lines represent conventional hydrogen bonds, while cation–pi interactions are rendered in yellow, pi–pi interactions in purple, hydrophobic interactions in white, and other interactions in pink.

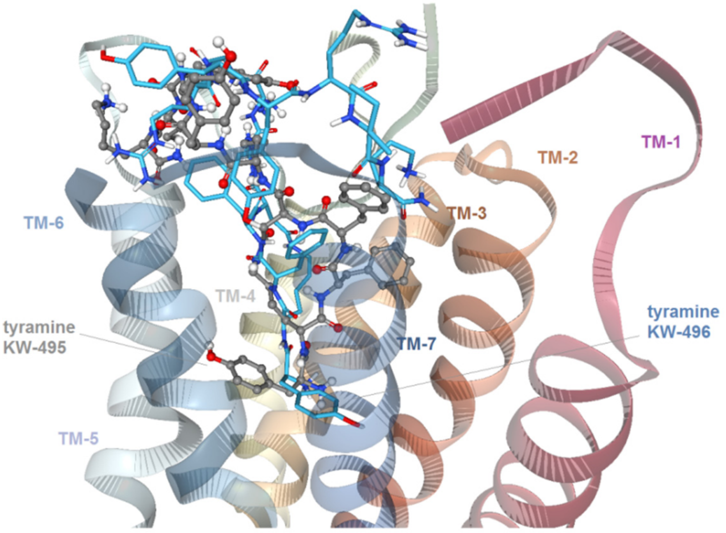

Figure S6. Overlay of **KW-495** and **KW-496** into KOP, obtained with PacDOCK web server (https://pegasus.lbic.unibo.it/pacdock/)

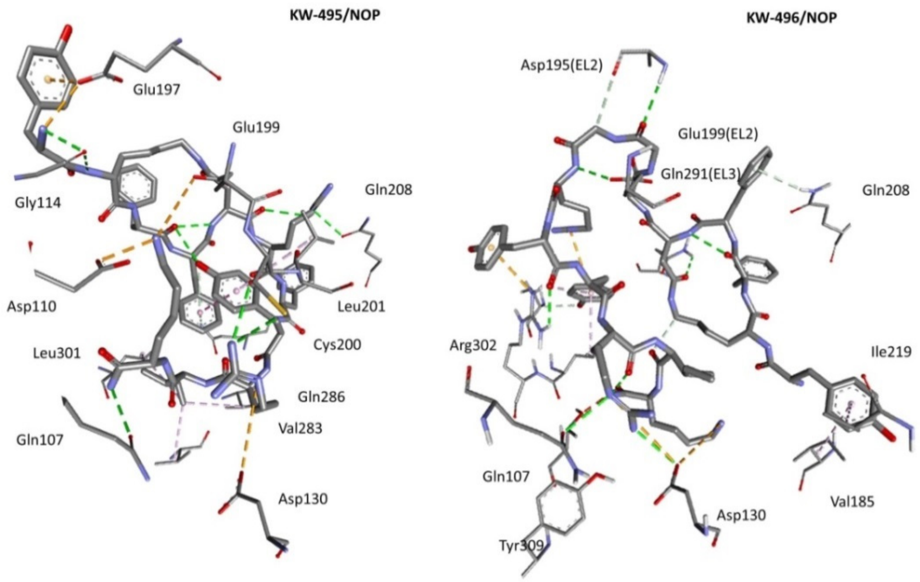

Figure S7. Specific ligand-receptor interactions obtained with Biovia Discovery Studio 2016 for **KW-495**/NOP and **KW-496**/NOP not discussed in the main text are resumed here. In **KW-495**, Tyr^1-c[D-Lys-Phe-Phe-Asp]-Arg^6-Tyr-Tyr-Arg-Ile-Lys^{11}-NH_2, the phenolic OH of Tyr^8 points towards Phe^{3}C=O forming an intramolecular H-bond, while its aromatic ring is stacked between Phe^{4}aryl (intramolecular pi-pi) and Leu201 (pi-alkyl); Tyr^7 is hosted within a pocket delimited by residues from the top of TM-5 and 6, and by residues of EL-3; the guanidine of Arg^6 is held in place by a H-bond with Gln208C=O and by a intramolecular H-bond with Asp^{5}C=O; D-LysNH is H-bonded to Gly114C=O; the Phe^{4}aryl group also interacts with Gln286$CONH_2$ (pi-H-bond). Asp^{5}NH is H-bonded to Phe^{3}C=O (intramolecular, 7-membered γ-turn). In **KW-496**/NOP, Gly^8 and Gly^7 interact with Asp195(EL-3). Few interactions contribute to stabilize the pose of the cyclopeptide: Asp^{5}β-carbonyl and Gln291 from EL-3 (H-bond), Phe^4 and Gln208 (pi-H-bond), and Tyr^1 phenol with Val185 and Ile219 (pi-alkyl). Receptor residue side chains are rendered in thick lines and the ligands in sticks; C is rendered in grey, N in blue, and O in red. Salt brides are rendered as dashed lines, dashed green lines represent conventional hydrogen bonds, while cation–pi interactions are rendered in yellow, pi–pi interactions in purple, hydrophobic interactions in white, and other interactions in pink.
